# On the Material Characterisation of Wind Turbine Blade Coatings: The Effect of Interphase Coating–Laminate Adhesion on Rain Erosion Performance

**DOI:** 10.3390/ma10101146

**Published:** 2017-09-28

**Authors:** Enrique Cortés, Fernando Sánchez, Anthony O’Carroll, Borja Madramany, Mark Hardiman, Trevor M. Young

**Affiliations:** 1 Aerox Advanced Polymers, 46185 Pobla Vallbona-Valencia, Spain; ecortes@aerox.es (E.C.); bmadra@aerox.es (B.M.); 2 Institute of Design, Innovation and Technology (IDIT), Universidad Cardenal Herrera-CEU, CEU Universities, 46113 Moncada-Valencia, Spain; 3 Irish Composites Centre (IComp), Bernal Institute, School of Engineering, University of Limerick, Limerick V94 T9PX, Ireland; anthony.ocarroll@ul.ie (A.O.); Mark.Hardiman@ul.ie (M.H.); trevor.young@ul.ie (T.M.Y.)

**Keywords:** wind turbine blades, rain erosion, coatings, leading edge protection, differential scanning calorimetry, peeling, pull-off, nanoindentation

## Abstract

Rain erosion damage, caused by repeated droplet impact on wind turbine blades, is a major cause for concern, even more so at offshore locations with larger blades and higher tip speeds. Due to the negative economic influence of blade erosion, all wind turbine Original Equipment Manufacturers (OEMs) are actively seeking solutions. In most cases, since the surface coating plays a decisive role in the blade manufacture and overall performance, it has been identified as an area where a solution may be obtained. In this research, two main coating technologies have been considered: In-mould coatings (Gel coating) applied during moulding on the entire blade surface and the post-mould coatings specifically developed for Leading Edge Protection (LEP). The coating adhesion and erosion is affected by the shock waves created by the collapsing water droplets on impact. The stress waves are reflected and transmitted to the laminate substrate, so microstructural discontinuities in coating layers and interfaces play a key role on its degradation and may accelerate erosion by delamination. Analytical and numerical models are commonly used to relate lifetime prediction and to identify suitable coating and composite substrate combinations based on their potential stress reduction on the interface. Nevertheless, in order to use them, it is necessary to measure the contact adhesion resistance of the multi-layered system interfaces. The rain erosion performance is assessed using an accelerated testing technique, whereby the test material is repeatedly impacted at high speed with water droplets in a Whirling Arm Rain Erosion Rig (WARER). The materials, specifically the coating–laminate interphase region and acoustic properties, are further characterised by several laboratory tests, including Differential Scanning Calorimetry (DSC), pull-off testing, peeling–adhesion testing and nanoindentation testing. This body of work includes a number of case studies. The first case study compares two of the main coating technologies used in industry (i.e., gel coating and LEP); the second case investigates the effects of the in-mould gel coating curing; and the third considers the inclusion of a primer layer on a LEP configuration system. Following these case studies, the LEP is found to be a far superior coating due to its appropriate mechanical and acoustic properties and the interface between the coating and the substrate is highlighted as a key aspect, as poor adhesion can lead to delamination and, ultimately, premature failure of the coating.

## 1. Introduction

**Figure 1:**
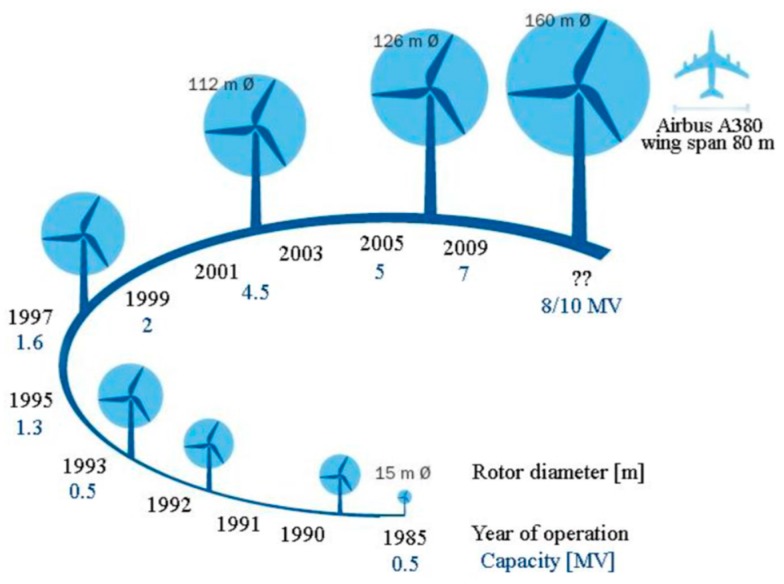
Size evolution of wind turbine blades [2].

The EU objective to cut greenhouse gas emissions by 80–95% by 2050 has severe implications for the energy sector [1]. By 2050, wind power will provide more electricity than any other technology in this sector. In the near future, electricity production requirements are to be almost emissions-free and the EU will encourage and facilitate the development of renewable and low-emission sources of energy. Many renewable technologies require further maturity to bring down costs. There is a need to improve existing technologies, such as wind energy, by increasing the size of offshore wind turbines to capture more wind energy. The offshore wind renewable energy community will potentially be the biggest contributor to meeting the EU objective and can supply substantial quantities of electricity with declining costs. The installation of very large wind turbines (10 MW and higher), will be necessary in pursuit of this. It is projected that wind turbines with increased rotor diameters will continue to be developed and installed (see Figure 1). In this case, wind turbine blades with a length of up to 90 m will be in operation in the near future, with increased tip speeds from 80 m/s to over 110 m/s. When considering the impact of rain droplets, the tip speed is a key contributor to erosion damage.

**Figure 2:**
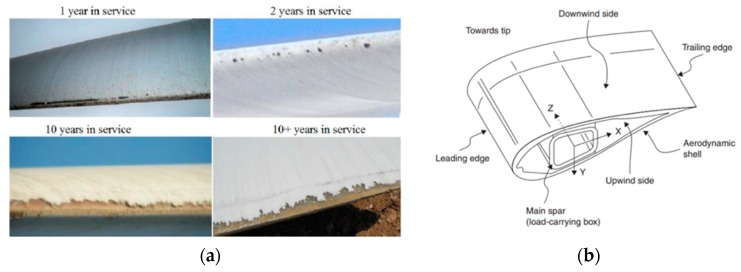
(**a**) Examples of leading edge erosion across a range of years in service [13]; (**b**) blade section with leading edge location where the rain erosion protection is critical.

Leading edge erosion of wind turbine blades has seen an intense increase in both damage initiation and the rate at which the damage progresses (see Figure 2). This is significant for offshore wind turbines with larger blades and higher tip speeds than onshore turbines. Among the consequences, erosion degrades the aerodynamic performance of the blade, leading to potential losses in the annual energy production, and may also compromise the structural integrity of the blade depending on the extent of erosion. Blade rain erosion is now identified as one of the major blade issues, affecting all wind turbine types [3]. This will undesirably impact the offshore wind industry, especially if erosion repairs are to be undertaken on offshore wind farms. Now that the offshore market is steadily increasing, this situation will pose substantial challenges. It is therefore key to maximise the performance and reliability of the composite technologies employed in manufacturing wind turbine blades. Due to the negative economic impact of blade erosion, all wind turbine Original Equipment Manufacturers (OEMs) are actively seeking solutions to this problem [4,5,6,7]. In most cases, the coating is the component being optimised as it plays a crucial role in both the blade manufacture and the overall performance of the blade. Rain erosion protection coatings have been proposed, tested and validated with particular industrial solutions [8,9,10,11,12,13], but the proposed solutions are still not as reliable as the wind energy industry requires. Rain erosion has thus become a scientific challenge for the wind industry since there are no well-defined methodologies to design coatings against rain erosion and it is unclear how to modify their properties depending on the location, weather conditions, etc. Moreover, there is a lack of consideration o; the coating design to be integrated into the blade manufacturing process.

In this work, an investigation into the mechanical characterisation of the multi-layered composite system, focusing on the coating–laminate interphase, is being undertaken. Firstly, the overall coating materials and processing technologies approach employed in the blade manufacturing are introduced. Secondly, we provide an overview of the liquid impact phenomena allowing one to identify how adhesion and erosion are affected by the shock wave caused by the collapsing water droplet on impact. Mechanical testing and rain erosion durability testing of the coatings were undertaken through accelerated rain erosion testing of a range of samples with processing parameter variations. The results and conclusions are validated and endorsed to optimise manufacturing and coating processes for blades into knowledge-based guidelines for leading edge coating material development.

## 2. Materials and Manufacturing Approach

**Figure 3:**
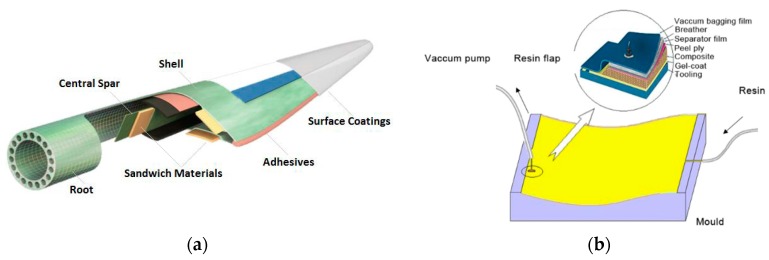
(**a**) Materials used in a typical blade cross section [15] and (**b**) surface-coated mould area and tooling materials used [15].

The large and ever-growing scale of modern blades has resulted in the widespread implementation of fibre-reinforced thermosetting polymer composite technologies due to their high specific strength and stiffness properties and fatigue performance. Glass–fibre composites such as glass-reinforced plastics (GRP) and carbon-fibre-reinforced plastics (CFRP) are appropriate materials for use in such applications. Their use opened up great prospects in the design and manufacture of future wind turbine blades due to the versatility offered in the material optimisation and design. Nevertheless, composites perform poorly under transverse impact (i.e., perpendicular to the reinforcement direction) and are sensitive to environmental factors such as heat, moisture, icing, salinity and/or UV. Blade manufacturers employ surface coatings to protect the composite structure from exposure to these factors [2,14]; see Figure 3a.

### 2.1. Infusion-Based Blade Manufacturing

**Figure 4:**
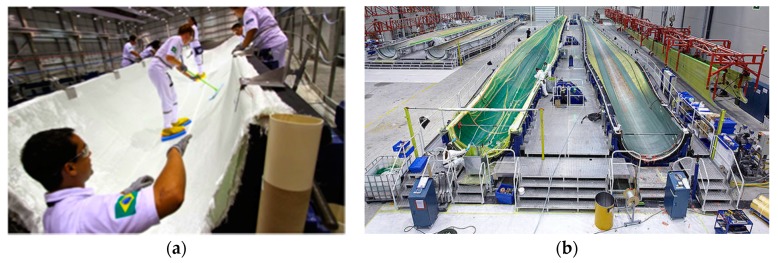
(**a**) Surface textile material application over the in-mould coating [6]; and (**b**) resin infusion process of a wind turbine blade [7].

Composite wind turbine blades can be manufactured using numerous processing methods. The two most common approaches are through pre-preg application or liquid composite moulding techniques (e.g., vacuum infusion or resin transfer moulding) [15,16,17,18]. Resin vacuum infusion (VI) is increasingly used in large-scale wind energy systems mainly due to cost savings and the suitability of the material processing and design. Each composite blade can be manufactured in half, in separate moulds, closed together and adhesively bonded. The principle of this moulding process involves firstly applying a release film on the inner part of the mould, followed by a thin layer of gel coating—see in Figure 3b. Subsequently, dry fibre reinforcements are placed over the coated area (shown in Figure 4a), followed by a peel ply, a separator film and a breather. Lastly, the whole system is enclosed in a bagging film, as shown in Figure 4b. Thus, the plastic film will function as the top part of a mould. Under these settings, the vacuum generated in the closed cavity allows the liquid resin to spread and impregnate the fibre reinforcements until complete saturation is achieved. Once the resin is cured, the plastic film, peel ply, separator film and breather are removed and the upper or lower mould parts are de-moulded—at which point the two blade component shells are finished [15].

### 2.2. Coating Technologies

**Figure 5:**
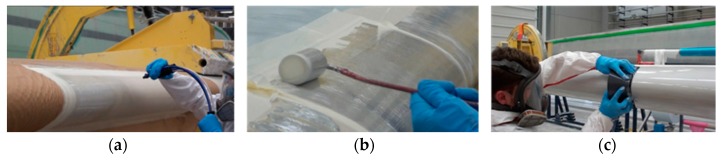
Leading edge protection application procedures, i.e., (**a**) spray; (**b**) roller; (**c**) trowel.

**Figure 6:**
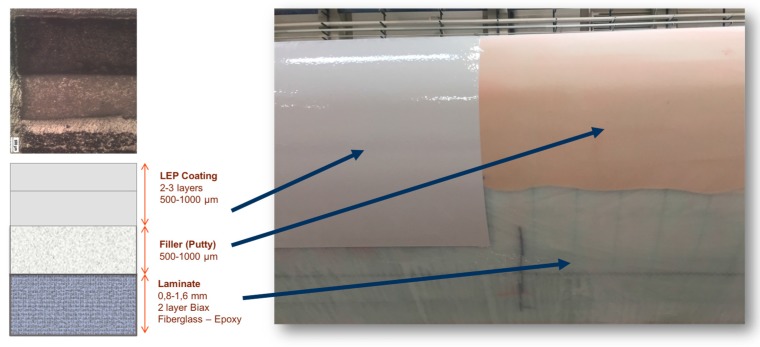
A particular leading edge protection system configuration on the blade surface.

**Figure 7:**
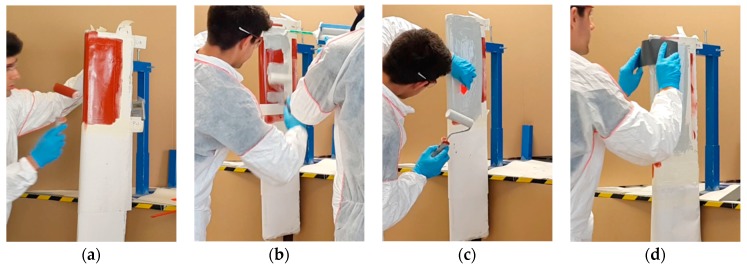
Leading edge protection service application with different techniques: (**a**) application of the primer layer; (**b**) application of the putty/filler/LEP material; (**c**) the thickness of the coating needs to be monitored closely; (**d**) the surface of the material is screeded down to ensure a flat, smooth surface following the blade contour.

In wind turbine blades liquid moulding manufacturing, two common surface coating technologies can be employed [13]:

- In-mould coatings use a similar material to the composite matrix substrate, i.e., epoxy/polyester. The in-mould coating plays a key role in the product performance of the whole blade part and is used in liquid composite moulding processes. The ease of integration into the blade manufacturing process makes it advantageous, as it simplifies and reduces the cost of applying the coating system. An appropriate in-mould gel coating needs to meet the blade processing window requirements. The finished product must be adequately bonded to the reinforcement, have an appropriate surface finish and provide long-term protection [18].
- A post-mould application, through painting or spraying, has different flexible materials to choose from, a polyurethane-based coating for example, see Figure 5. Post-mould application is typically used to apply Leading Edge Protection (LEP) in locations where the threat of rain erosion is a concern. Industrial processes state that LEP systems can be outlined as a multi-layered system, where some manufacturers include a putty layer between the laminate and the coating. Some manufacturers also include a primer layer under the coating and over the putty to improve adhesion. Depending on each industrial solution, the inclusion of interfaces may accelerate erosion by delaminating between layers (to be discussed further in the next section)—see Figure 6. Applications with fewer coating layers are recommended because of the robustness of the process and the reduction of interfaces. The coating application procedure is designed with the final material properties in mind (i.e., thickness, number of coating layers, surface roughness, temperature, humidity, viscosity, processing time, curing time, etc.)—see Figure 5. Specific post-mould application methods and materials are similarly employed when repairing a damaged area, during a service or as part of a prevention maintenance programme. The repair of the leading edge damage is most frequently achieved through the unsophisticated application of a primer-based layer and putty materials, smoothed over, and then cured to generate a new uniform and smooth surface finished to the affected blade zone—see Figure 7. The coating manufacturer, however, can only guarantee the performance of such materials when applied in very specific environmental conditions.

In all the above mentioned cases, optimal interface adhesion between the surface coating system and the composite substrate laminate is necessary for sufficient mechanical performance and to match both systems’ properties in an integral solution, as will be analysed in the next sections.

## 3. Modelling of a Liquid Drop Impact on Wind Turbine Blades

Well-recognised rain erosion prediction models and case studies can be found in the literature in order to determine which coating factors affect erosion performance the most [9,10,11,19,20,21]. The rain erosive wear rate is dependent on a number of factors such as impact velocity, impingement angle, target material, droplet size, frequency of impact, etc. An essential aspect of understanding how erosion is caused on the coating material is to consider the physical effects initiated by the impingement of the liquid droplets upon the material surface. The analysis of the behaviour of a single water drop’s impact is a meaningful starting point for investigating the multiple impact sequences that produce leading edge erosion [11,22,23].

### 3.1. Liquid Impact Phenomena Affecting Erosion Failure

**Figure 8:**
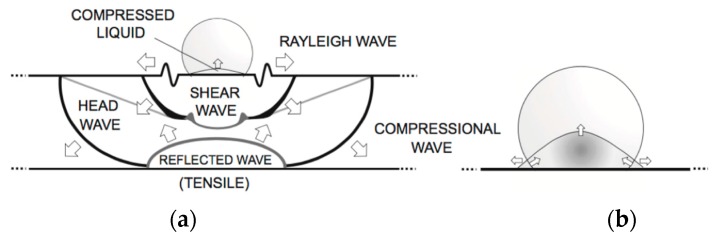
Liquid droplet–solid surface impact interaction, showing shockwave behaviour (from [20]). (**a**) Illustrates the three waves that develop following the droplet collision; (**b**) shows the lateral jetting upon movement of the contact boundary ahead of the shock wave in the drop initiating a release wave across the solid surface.

The analysis of erosion damage caused by rain droplets is based essentially on the static concept of direct impact on a rigid surface, although evidence shows that the damage is in fact a dynamic event resulting in the propagation of shock waves—see Figure 8. As the water droplet impinges on the surface at a normal angle, two wave fronts are created with the longitudinal compressional normal stress wave preceding a transverse shear wave. The impact gives rise to a third wave due to the water droplet deformation itself, called the Rayleigh wave, which is confined to the surface of the target and contains approximately 2/3 of the collision energy [20]. The pressure generated on impact is referred to as the water-hammer pressure and the magnitude varies depending on the acoustic properties of the target material and the liquid [24]. The maximum pressure does not occur at the epicentre of impact at the instant of first contact but at some delayed time in a ring around the midpoint at a location where the contact circle edge is reached by the initial shockwave generated by the impact [25]. Maximum shear stresses are observed on these radial locations and have a very short duration compared with the central compressional pressures. The duration of the impact pressure on the surface is directly related with the radius of the droplet. The erosion failure can be initiated by a local imbalance of tensile and shear stresses in regions that may be outside the direct impact area.

**Figure 9:**
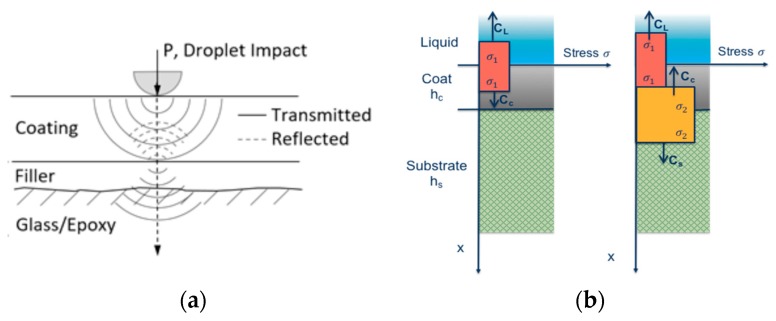
(**a**) Standard blade structure with a filler intermediate layer; (**b**) normal shockwave propagation depending on acoustic impedances in two consecutive time instants.

The post-impact shock wave also propagates through the multi-layer system materials and depends on the elastic and viscoelastic responses, the surface preparation, coating application and the interactions between layers. The understanding of these interactions through the mechanical modelling is limited but thought to be of key significance—see Figure 9a. Upon contact with the coating, two different wave fronts travel into the liquid and coating, respectively, as shown in Figure 9b. The normal incident wave front in the coating advances towards the coating–substrate interface, where a portion of the stress wave is reflected back into the coating and the remaining part is transmitted to the blade substrate system. Due to this reflection, a new wave is now advancing in the coating with a different amplitude depending on the relative magnitude of the acoustic impedances of the coating and substrate [23]. In this case, assuming a uni-dimensional and elastic approach, the amplitudes of the stress waves can be approximated by the following relations:

(1) $$\frac{\sigma_{R_{LC}}}{\sigma_{I_{LC}}}=\frac{Z_{L}-Z_{C}}{Z_{L}+Z_{C}}\;;\;\frac{\sigma_{T_{LC}}}{\sigma_{I_{LC}}}=\frac{2Z_{C}}{Z_{L}+Z_{C}}$$

(2) $$\frac{\sigma_{R_{CS}}}{\sigma_{I_{CS}}}=\frac{Z_{C}-Z_{S}}{Z_{C}+Z_{S}}\;;\;\frac{\sigma_{T_{CS}}}{\sigma_{I_{CS}}}=\frac{2Z_{S}}{Z_{C}+Z_{S}},$$

where Z=ρC is the impedance of the material, ρ is the density and *C* the elastic wave speed (the speed of sound of the medium). *Z_L_*, *Z_C_*, and *Z_S_* are the elastic impedances of the consecutive materials (i.e., in our problem they are the liquid (_L_), coating (_C_), and substrate (_S_) layers). $\sigma_{I_{LC}},\;\sigma_{R_{LC}}$, and $\sigma_{T_{LC}}$ are the amplitudes of the normal incident, reflected and transmitted stress waves, respectively, at the surface (Liquid–Coating interface) and $\sigma_{I_{CS}},\;\sigma_{R_{CS}}$, and $\sigma_{T_{CS}}$ the ones at the Coating–Substrate interface.

**Figure 10:**
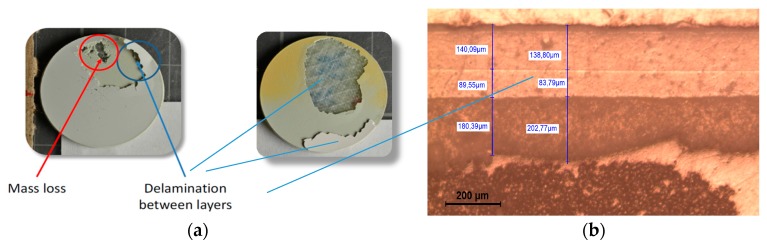
(**a**) Two different types of erosion failure: pits and cracks that progress with mass loss caused by direct impact and stress on surface (left) and delamination indirectly caused by the interface stresses (right); (**b**) Cross section of a multi-layered system. Two consecutive coating layers and coating–substrate interfaces in which delamination tend to appear upon impingement.

Moreover, this impact shock wave is also reflected wherever the acoustic impedance properties differ locally, so microstructural defects, such as voids, blisters and lack of adhesion, play a key role on the degradation of a particular coating. Hence, indirect damage by delamination may occur at the interface boundaries between material layers, caused by the propagation and interaction of the compressional waves from the impact of water droplets, as shown in Figure 10a,b.

### 3.2. Identify Suitable Materials for Rain Erosion Coating Protection

The capability of the coating to transfer wave energy in the multi-layered system can influence the erosion damage. Stress reflections oscillate repeatedly through the coating and substrate structure until dampened out by the materials’ properties, to reduce the energy of the initial shockwave [25]. Numerous consecutive impact droplets result in the interaction of the reflected waves from microstructural discontinuities and positive wave interferences which produce tensile stresses with an amplitude that can be greater than the dynamic ultimate strength of the material. With coatings, the relative acoustic impedance effect is known to reduce the stress when transmitted from the coating to the substrate layer [10,23]. Table 1 shows the impedance properties of selected candidate materials treated in previous sections. In the table *Z_L_*, *Z_C_*, and *Z_S_* denote the impedances of the liquid, coating and substrate, respectively. Their ability to reduce or increase the incident impact stress wave is quantified via Equations (1) and (2) and allows one to identify, as a first approach, the suitable material combinations.

In a rigid composite substrate typically used in wind turbine blades (i.e., GRFP glass fibre reinforced epoxy or polyester), there are two main coating technologies as introduced earlier: in-mould gel coatings and post-mould LEP coatings. The in-mould gel coatings, usually employed for the whole blade protection, are rigid, brittle and have a high modulus due to the epoxy or polyester polymer-based formulation. Owing to their high acoustic impedance, these gel coatings are able to moderate the transmitted stress to the substrate and to the coating surface. On the other hand, the post-mould coatings are usually developed for the Leading Edge Protection (LEP) where the rain droplet impact energy is greater, mainly due to the higher tip speeds. These LEP elastomer material coatings are formulated with low macroscopic elastic modulus, high ultimate strain and high resilience that will reduce the stress at the impact surface and dampen the stress waves, ensuring that the recovery time of the material is rapid and the energy is dissipated quickly (depending on the dynamic properties and the thickness). It is important to remark from Table 1 that the transmitted stress waves are reduced significantly from the liquid to the coating and, on the other hand, amplified from the coating layer to the substrate, which in fact it is not a problem due to the higher mechanical capabilities of the laminate. These materials store energy at a reasonably low level of stress (at a value lower than the fracture strain) but need to be defined considering the appropriate adhesion between the coating and the substrate and their relative impedances. In order for the system to lower its total free energy, pits and micro-cracking take place as a major high rate recovery mechanism. Depending on the mechanical properties of the material, the damage progresses with pits and cracks on the surface yielding mass loss and propagate by the usual fatigue characteristics. It is important to note, as it was introduced in previous sections and will be exposed in this work, that intermediate layers of putty fillers or primer materials may develop complex stress wave interactions. Optimising the acoustic properties of each layer so that they work together, while limiting delamination, should extend the lifetime under repeated impacts.

### 3.3. Erosion Lifetime Prediction Modelling

**Figure 11:**
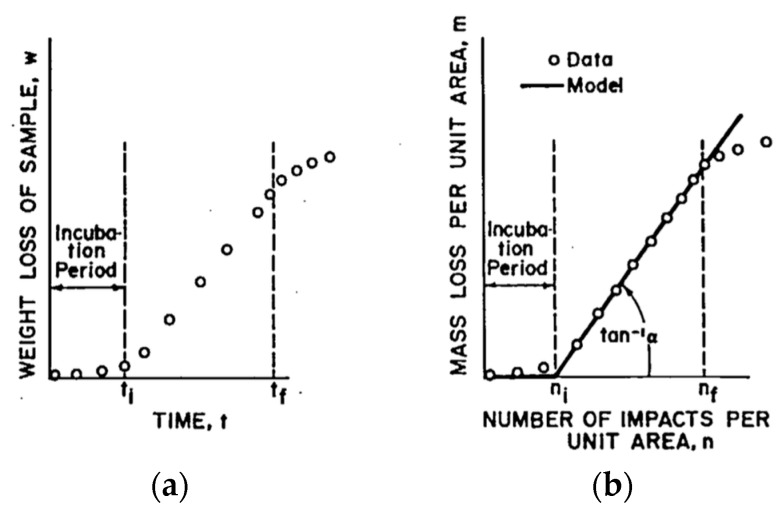
(**a**) Representative evolution of weight loss on experimental rain erosion testing samples, from [23]; (**b**) lifetime prediction model defining the incubation period and mass removal rate.

The progression of erosion can be experimentally measured in a number of ways. One method is in terms of the average erosion depth versus time [26] or mass loss versus time (directly related to the number of impacts) [10]—see Figure 11. There is initially an incubation period in which damage progresses without perceptible change in the material weight loss. After a sufficient amount of fatigue degradation has accumulated, the material tends to lose mass with a constant erosion rate. This marks the end of the incubation period and a steady mass loss period begins, where the weight loss varies nearly linearly with time. Figure 11 depicts the modelling proposed in [10] and is also considered as the standard to quantify the damage ASTM G73-10 (Liquid impingement erosion using rotating apparatus) [27]. This analytical model has been widely referenced in flight applications [20] and recently applied successfully to wind turbine blades, as described in [28]. The model quantitatively predicts the erosion of coated materials under the previously untested conditions.

In order to predict the incubation time and the mass removal rate, the stress history in the coating and in the substrate has to be identified analytically or numerically. It is affected by the shockwave progression due to the vibro-acoustic properties of each layer, and by the frequency of the repeated water droplet impacts. Fatigue life of the material is calculated using an equivalent dynamic stress with a semi-empirical approach and depends on the ultimate tensile strength and other relevant properties of both the coating material and substrate. The model can be applied to estimate the stress at different locations through the thickness, i.e., the coating surface or at the coating–substrate interface. Nevertheless, it is assumed that the bond and adhesion of the boundary interface is ideally perfect, so the modelling does not account for the microstructural imperfections and lack of adhesion of such interfaces.

**Figure 12:**
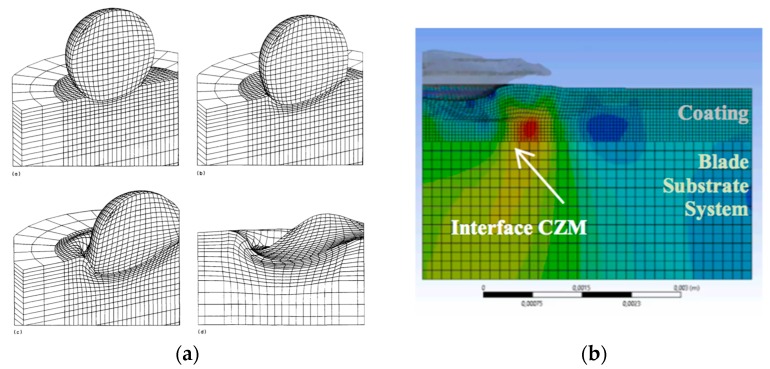
(**a**) 3D FEM modelling of a water droplet impact in a thick polymer solid, from [22]; (**b**) numerical modelling of normal stress due to rain droplet impact. Interface CZM defines the failure resistance between contact layers, as in [29].

The appropriate development of numerical modelling of rain droplet impact can also be used to compute the stress history in the material [19,22]—see Figure 12a. In [29] it is proposed to incorporate cohesive zone modelling (CZM) between layers in the numerical modelling of the droplet impact—see Figure 12b. With both analytical and numerical approaches, it is necessary to characterise the failure resistance of the multi-layered system interface boundaries in order to use erosion lifetime prediction models such as the one described previously.

## 4. Case Studies: Effect of Interface and Adhesion Issues on Rain Erosion Performance

**Figure 13:**
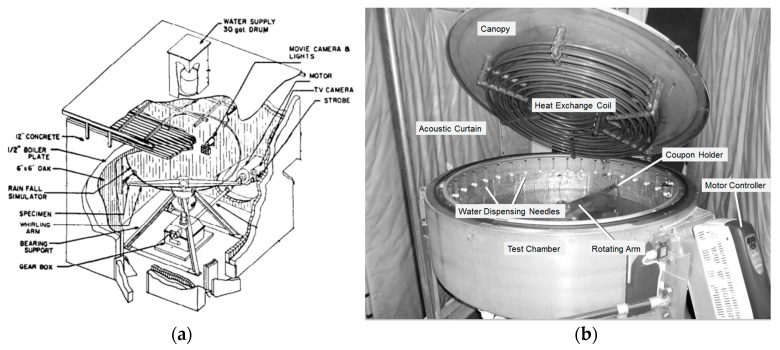
(**a**) Rain test facility at University of Dayton Research Institute, developed for aerospace applications [13]; (**b**) whirling arm rain erosion facility (WARER) at University of Limerick [31].

Due to the absence of suitable rain erosion testing standards within the wind sector, the industry looked to the aerospace sector [13]. Evaluations can typically be performed using a rotating arm rain erosion test to ASTM G73-10 (liquid impingement erosion using a rotating apparatus, shown in Figure 13) [27]. Assessment is normally undertaken through a combination of mass loss measurements and surface characterisation. Interpretation of mass loss data can be difficult as it does not distinguish between erosion depth and the area of damage. Another difficulty concerns the inability to directly correlate laboratory testing and in-service erosion. Surface topography of test coupons (measured using a confocal laser scanning microscope) has also been used as a means to characterise damage and provide a partial correlation between different test facilities [30,31,32]. Despite the inherent complexities of simulating real-life erosion in a laboratory environment, such testing has been shown to be very helpful in rating rain erosion resistance of different materials and in characterising the induced damage.

The objective of the current research is to assess the relationship between the coating–laminate interphase characterisation and the material’s resistance to erosion damage, through mass loss measurements.

The rain erosion testing of this work was conducted in the University of Limerick’s whirling arm rain erosion facility (WARER, pictured in Figure 13b), which is described in [31]. The test procedure, which is based on ASTM G73-10 [27], evaluated a range of coatings at an impact speed of 135 m/s in an artificially generated rain field comprising of 2 mm diameter droplets, produced by 36 needles at a rate of approximately 25.4 mm/h. The evolution of damage was monitored through mass loss and visual examination of the specimen surfaces.

### 4.1. Comparison of Distinctive Polymer-Based Coating Technologies

**Figure 14:**
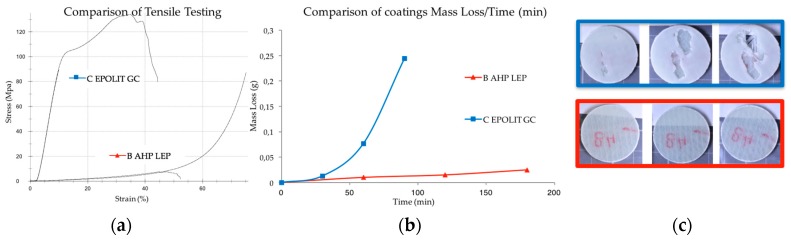
(**a**) Tensile test of a rigid in-mould coating (EPOLIT GC) compared with an elastomeric LEP coating (AEROX AHP LEP); (**b**) Average mass loss versus time for the two different coating technologies; (**c**) Images of erosion damage after 30, 60 and 90 min. (EPOLIT GC, upper series in blue and AEROX AHP LEP, lower series in red).

A first experiment was focused on evaluating the erosion resistance of two coating types. The coupons were comprised of a coating (0.3 mm thick) applied to two layers of biaxial epoxy–GF (1.4 mm thick). The circular coupons had a diameter of 27 mm. The two coating types were compared using the same substrate: first, an in-mould gel coating prototype (EPOLIT GC E 13X, range of products), which has an epoxy-based formulation and second, a post-mould leading edge protection coating prototype (AEROX AHP LEP 900, range of products), which has an elastomeric-based formulation. The testing results of the eroded samples are shown in Figure 14.

No surface damage is assessed on the elastomeric coating samples during the testing period as was predicted based on its acoustic properties, see Table 1. Conversely, focusing on the in-mould coating performance (EPOLIT GC), small cracks and pits are apparent on the specimen surface after 30 min. At this point the incubation period has ceased and severe delamination failures occur in the damaged areas in the subsequent intervals. The damage rapidly reveals the bare laminate; see Figure 14c.

This type of erosion failure that yields coating delamination has premature and catastrophic consequences for the composite laminate and has to be assessed for both techniques. As a first approach, it is evident that the samples manufactured with a more rigid material performed worse in regard to erosion compared to those that had a low modulus of elasticity and high strain rate deformation capability.

### 4.2. Effect of Curing Conditions of In-Mould Blade Coatings on Erosion Performance

In this section, the in-mould curing conditions are investigated with a view to assessing how they affect erosion performance. It is very important to determine the effect of surface coating curing time on the dynamic behaviour of the resin when infusing a fabric [33,34]. In a previous work developed by the authors [35], a mixed numerical/experimental technique based on artificial vision is used to estimate the induced effect of the surface in-mould coating curing in the laminate impregnation and the flow front advance during filling.

**Figure 15:**
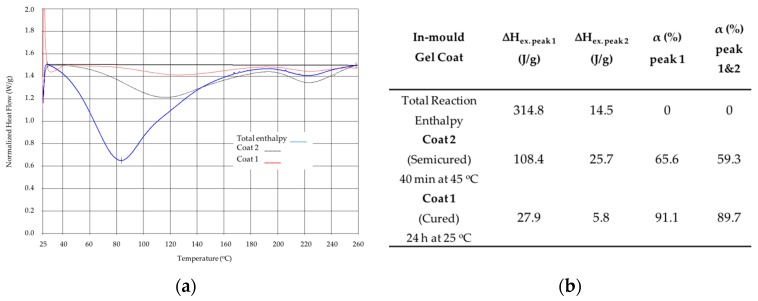
(**a**) DSC measures reaction enthalpy; (**b**) degree of curing conversion (α) measured data.

Two different curing conditions for the in-mould gel-coat EPOLIT GC are used in order to generate differences in the impregnation and flow advancement of the epoxy resin in the dry laminate preform during filling. The whole part is completely cured in both cases before demoulding. The substrate laminate is manufactured in both cases with two-layer biaxial glass fibre (1.4 mm thick) and the in-mould coating layer is defined as 0.3 mm, as in the previous case. Coat 1 and Coat 2 were previously characterised by performing a measure of the degree of conversion of the polymerisation reaction of the polymer matrix. The degree of conversion (α) is obtained by measuring the residual enthalpy ΔH (J/g) using Differential Scanning Calorimetry (DSC). Coat 1 is cured for 24 h at 25 °C, which is considered to be a full cure (89.7% cured), whereas Coat 2 is cured for 40 min at 45 °C, which is considered to be semi-cured (59.3% cured). The samples are quantified and outlined in Figure 15.

**Figure 16:**
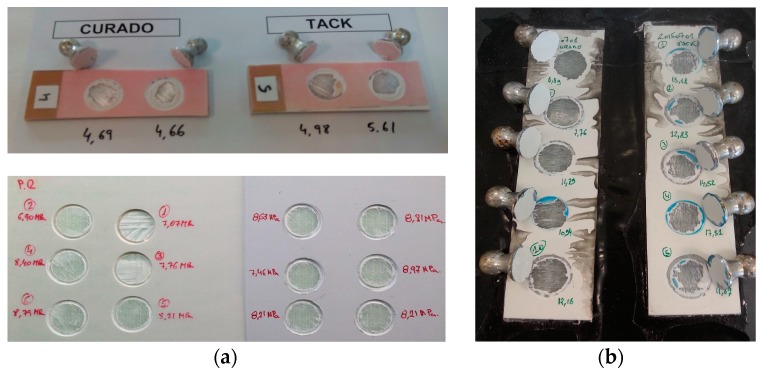
(**a**) Pull-off strength testing of composite laminates used for coating adhesion. Images of the failure in the laminate (**b**).

**Figure 17:**
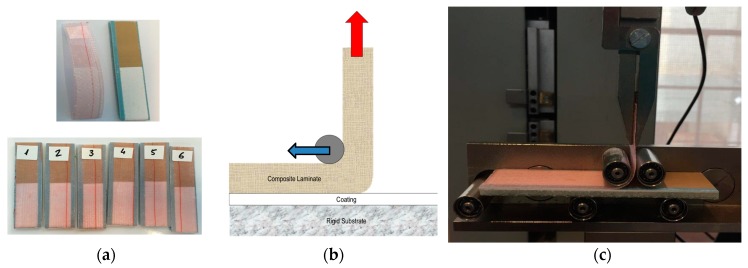
Developed peeling testing for interphase coating-laminate adhesion response quantification. (**a**) Developed specimens; (**b**) forces on testing; and (**c**) peeling testing system.

**Figure 18:**
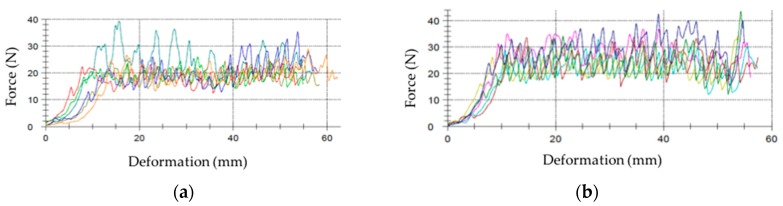
(**a**) Force of failure for interphase adhesion testing: Coat 1 (Cured), average value of 19.3 N (averaged across six samples, plotted in different colors) and (**b**) Coat 2 (Semi-cured), average value of 25.1 N (averaged across six samples, plotted in different colors).

The typical mechanical testing used in the wind turbine industry for material qualification is shown in order to assess the macroscopic behaviour of the laminates and how it is influenced by the coating curing conditions. In Figure 16, pull-off strength testing of the samples shows different cases in which the failure is in the composite laminate, and hence the ability of the coating to assure the required target strength. No information regarding the interphase strength is given in cases where the failure does not take place in the coating or in the interphase, but it does indicate a limit value. Moreover, there is a lack of information in the literature regarding the curing effect on the interphase. Figure 17 shows a specially developed peeling test for interphase coating–laminate adhesion response quantification. The samples are moulded over a rigid substrate, where the coating is bonded with a special adhesive and hence the differences on the adhesion laminate-coating depending on its curing can be measured. Figure 18 specifies the failure load for peeling interphase adhesion testing. Coat 1 (Cured) has an average value 19.3 N and Coat 2 (Semi-cured,) of 25.1 N.

**Figure 19:**
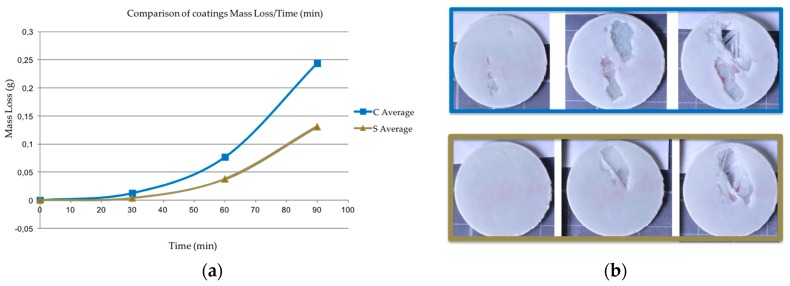
(**a**) Average mass loss versus time for two different coatings: Coat 1 (Cured, C in blue) and Coat 2 (Semi-cured, S in Green); (**b**) Images of surface and delamination damage after 30, 60 and 90 min of testing. Upper series for Coat 1 and Lower series for Coat 2.

These results correlate well with the rain erosion tests, as shown in Figure 19. The semi-cured coating shows better erosion performance. The incubation time is delayed and also decreased the erosion rate. In the depicted images it can be observed that delamination is less severe owing to its greater adhesion at the interface.

**Figure 20:**
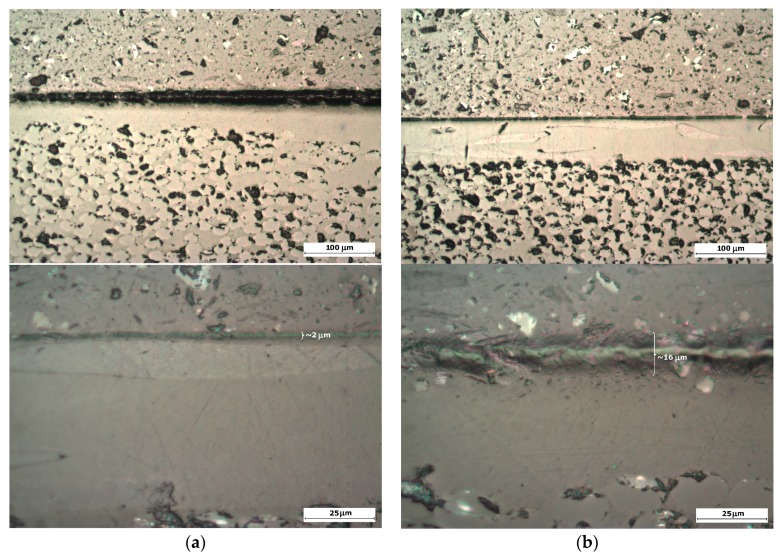
(**a**) Microscopy samples for interphase chemical adhesion: Coat 1, (Cured) and (**b**) Coat 2, (Semi-cured). Upper images magnification 100×, lower images are 400×).

In Figure 20, it can be seen how the semi-cured coating (curing conversion α = 59.3% instead of α = 87.9% for the same epoxy-based polymer, EPOLIT GC) defines a broader interphase area in the epoxy resin infused GF laminate due to a higher chemical bonding between the resin and the gel coat. This interface layer thickness may vary through the specimen, but clearly defines a local variation of properties on the contact region.

**Figure 21:**
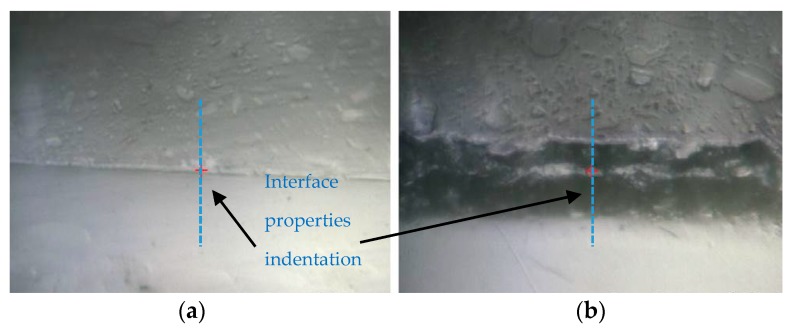
Layout of the indentation for measuring bulk properties of the gel coating and interphase properties of the two cases of curing. (**a**) Coat 1 (Cured) and (**b**) Coat 2 (Semi-cured).

In order to explore a local variation of the acoustic impedances due to the interface layer in both cases, nanoindentation testing is done. First, the bulk properties of both gel coats and composite GFRP matrix materials are obtained; see Table 2 and Figure 21. Ten indents are carried out into each material, Coat 1 and Coat 2. The maximum indentation depth was set to 2 µm and an indentation strain rate of 0.05/s was used. The maximum indentation load was held for a total of 120 s in order to allow time-dependent deformation to diminish prior to the critical unloading segment of the test [36]. It was ensured that the indents were positioned far from the coating–matrix interface and from the previous indent impressions. The results for the bulk properties for both samples are equal, where the gel coating has a slightly higher modulus and hardness than the GFRP epoxy-based matrix. It is important to note that the indents are not applied on or near the fibre reinforcement of the GFRP laminate, only in the matrix, so the macroscopic properties of the composite laminate may vary.

**Figure 22:**
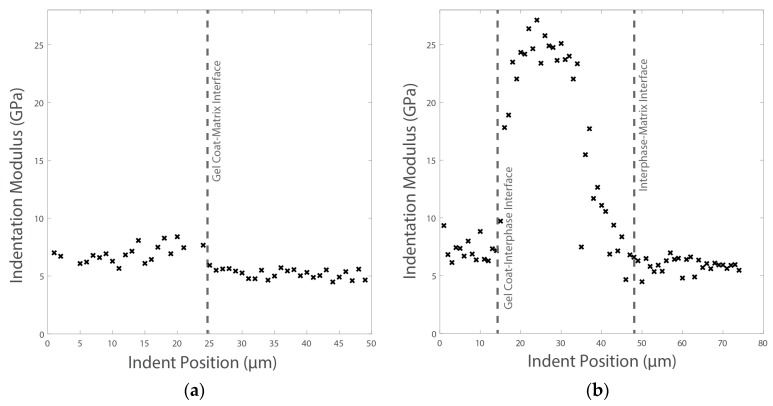
Two series of indents across the interface for the two samples of in-mould Gel Coating and the epoxy-based matrix of the GFRP laminate with different curing conditions: (**a**) Coat 1 (Cured) and (**b**) Coat 2 (Semi-cured).

In subsequent testing, lines of indents are carried out across these interfaces through the thickness; see Figure 21. The indents are applied to a maximum depth of 100 nm with a spacing between each indent of 1 µm. For sample Coat 1 (Cured), a distinct interface is detected where the indentation modulus for the EPOLIT GC gel coating is slightly larger than that of the GFRP composite epoxy-based matrix material; see Figure 22. However, for the Coat 2 (Semi-cured) sample, a clear interphase is present between the materials, where the interphase has a much larger stiffness than either material. This result correlates well with the erosion testing and also with the acoustic impedance variation due to the interphase.

As can be observed in Table 3, different configurations of gel coating candidate materials are considered. A material combination of EPOLIT GC, cured gel coat, Coat 1, with a GFRP substrate, yields different relative acoustic impedance than if the GFRP matrix is considered as a substrate. This last option is unrealistic and has not been considered since it does not account for the effect of the fibre reinforcements. It is shown in terms of completeness due to the indentation testing.

A very interesting result concerns the improved acoustic effect of the Coat 2 interphase, i.e., Semi-cured. The surface coating layer is defined by the EPOLIT GC gel coating bulk properties, which are the same for both Coat 1 and Coat 2, and were obtained far from the interface. In this case, the first subsequent substrate layer is considered to be defined by the Coat 2 interphase. It can be appreciated how the relative impedances from the Gel Coat–Coat 2 interphase layers generate and attenuate the reflected and transmitted waves from the coating to the first substrate layer and even larger from this one to the GFRP laminate. That result correlates well with the delayed incubation time obtained from the erosion testing and observed in Figure 19.

The key points to take from this case study are that the adhesion of the coating to the substrate is of paramount importance for the rain erosion resistance of the material. The coating, which was only partially cured before the fibre reinforcement was inserted, out-performed the coating, which was fully cured, in all aspects of the test.

### 4.3. Effect of Primer on the Performance of Leading Edge Protection (LEP) Coatings

**Figure 23:**
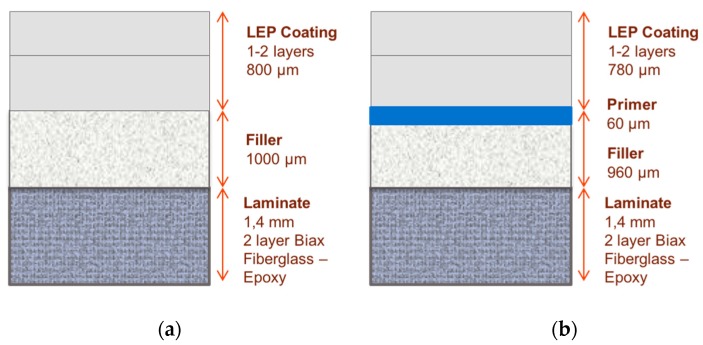
(**a**) Leading edge protection coating with an intermediate filler layer; (**b**) additional primer layer included to improve adhesion to the substrate.

In this section, a post-mould coating system configuration that includes a filler layer between the surface coating and the composite laminate is assessed. The performance of this configuration was compared to a comparable system that includes an additional primer layer to improve the contact adhesion of the coating to the substrate, as depicted in Figure 23.

The two configurations make use of a similar prototype LEP material (AEROX AHP LEP900, a range of products) with the substrate defined by an intermediate filler layer prototype (EPOPUR MS900, a range of products). The substrate (from the EPOPUR MS range), and the composite laminate, in both cases, is a two-layer biaxial Glass Fibre laminate (1.4 mm. thick). The primer layer included in one of the configurations is based on a prototype material (EPOLIT PR100, range of products). This configuration is not optimised in terms of everlasting lifetime, so erosion rate is not an issue in this case. The objective is defined for experimental rapid testing purposes and delamination failure analysis.

**Figure 24:**
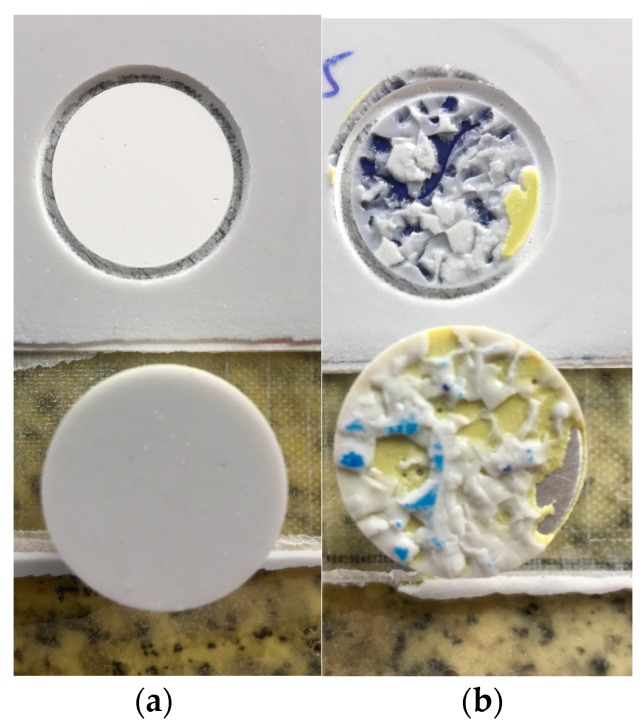
Pull-off strength testing of specimen laminates used for LEP coating adhesion: (**a**) adhesive failure with no-primer intermediate layer application; (**b**) cohesive failure with primer application (the dolly adhesive effect can also be observed).

**Figure 25:**
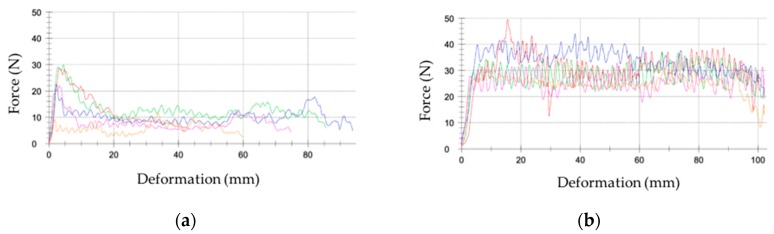
(**a**) Force of failure for interphase adhesion testing: LEP coating configuration with no-primer application, average value of 9.45 N and (**b**) LEP coating configuration with intermediate primer layer, average value of 29.31 N.

In Figure 24, pull-off testing of the samples shows the adhesive failure for the no-primer configuration (with a value of 5.6 MPa) and the cohesive failure (6.77 MPa) of the specimens that include the primer layer (b). The LEP in the no-primer coupon has been cleanly pulled off, whereas the primer coupon has not come off as easily. Figure 25 demonstrates the improved interphase coating–laminate adhesion response when the primer layer is included, with a force load for peeling with a value of 29.3 N (averaged across five samples), versus a value of 9.45 N for the no-primer configuration. It is clear that the primer significantly improves the adhesion of the LEP to the filler.

**Figure 26:**
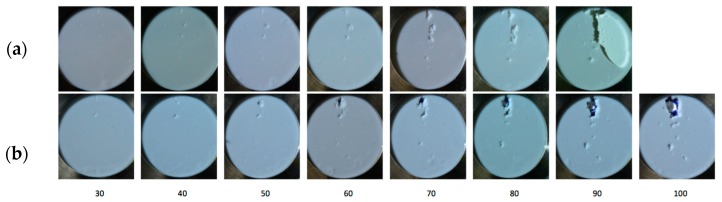
Images of surface and delamination damage after time interval (in minutes) of testing: (**a**) LEP coating configuration with no-primer application; (**b**) LEP coating configuration with intermediate primer layer.

These testing results correlate with the rain erosion tests, as shown in Figure 26. It can be observed in both cases how the erosion failure advances from the surface through the multilayer system thickness until it reaches the laminate. The incubation time (start of perceptible erosion) is outlined and can be quantified similarly in the pictures. The inclusion of the primer layer avoids delamination owing to the increase in fracture energy revealed by the peeling testing values. Delamination occurs only in the first configuration where no primer is included and hence, worse chemical bonding is achieved.

Complementary nanoindentation tests are carried out on the primer application configuration for more precise local interface discontinuities characterisation. The nanoindentation settings are as outlined in Section 4.2. First, a series of indents are created far from the interface on the LEP, primer and filler materials in order to calculate their bulk properties. The LEP and filler are indented to a depth of 5 μm, while the primer material is indented to a depth of 2 μm to avoid boundary effects due to its narrow width. The results for each constituent are shown in Table 4, highlighting the distinct properties of each layer.

**Figure 27:**
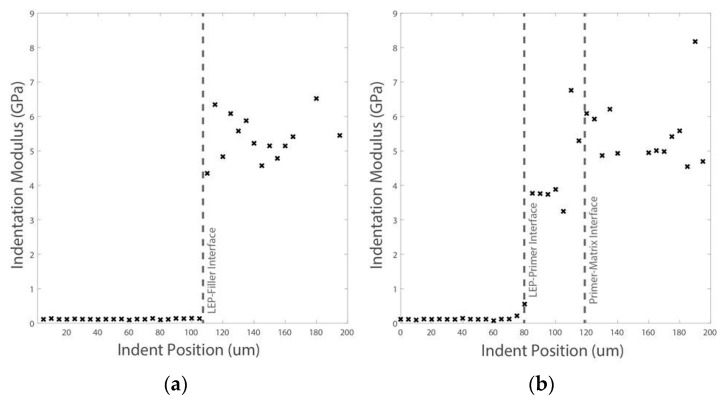
Series of indents across leading edge protection coating configuration with two distinctive interfaces. (**a**) LEP–filler interface and (**b**) LEP–primer–filler interface.

In addition, two lines of shallow (500 nm) indents across the interfaces of the three materials are implemented. Each line contained 40 indents, which were equally spaced by 5 μm, a total line distance of 200 μm. The resulting indentation moduli are shown in Figure 27 for Lines 1 and 2.

While a distinct change in modulus is apparent for the LEP–primer interface, the slightly lower modulus of the thin primer layer (compared with the filler) can be seen for the primer–filler interface. This result reveals a similar acoustic impedance on the primer–filler interface and also shows how the primer matches the filler acoustic impedance without pronounced discontinuities.

In Table 5, the relative acoustic properties are quantified for the different combinations of material candidates for the multi-layered system. It can be observed that the filler layer inclusion and even the primer layer does not negatively influence the reflected and the transmitted waves to the LEP compared to the direct application of the LEP over the GFRP laminate. Moreover, considering the primer layer as a first substrate layer over the subsequent filler layer, there is a reduced value for the reflected and transmitted stress waves. These results correlate well with the similar erosion incubation time observed in both configurations (with and without a primer) in the rain erosion testing summarised in Figure 19 for the LEP configuration.

The key result is that the inclusion of a primer layer, in this case, does not negatively influence the rain erosion performance of the coating systems. The adhesion of the coating to the substrate has been significantly improved, which, in turn, reduces the opportunities for delamination to initiate, offering a more reliable solution. Further optimisation of the primer material could improve the rain erosion performance of the entire system.

## 5. Conclusions

The development of new coating systems, with an aim to diminish the rain erosion damage in wind turbine blades, requires knowledge-based tools for erosion lifetime prediction and to identify suitable coating and composite substrate combinations. This research has been directed into the coating–laminate interphase adhesion characterisation in order to effectively predict rain erosion performance. The experimental work involved accelerated rain erosion testing, pull-off testing, peeling–adhesion testing and nanoindentation testing of individual coating configuration cases. The rain erosion testing results correlated well with the mechanical adhesion characterisation in all cases. In a first case, a low-modulus elastomeric material used for leading edge protection showed better response than a rigid gel coating. Their related impedances with a GFRP composite laminate were determined with a simplified approach. A second configuration case showed that samples manufactured with a higher degree of curing (as determined using DSC), were out-performed by those that had a lower degree of curing due to a more rigid and broad coating–laminate interphase during the in-mould curing. Another set of experiments, based on the typical LEP configuration, focussed on the effects of including a primer layer with a filler material within a multi-layer system. It was determined that the inclusion of a primer layer drastically improves the adhesion performance between the coating and the filler substrate and consequently avoids the erosion failure by delamination. Moreover, the incubation time of the erosion damage was not appreciably affected by the inclusion of the primer layer.

In this work, referenced simplified models were used to correlate the droplet impact shockwave stress history on the coating with the relative impedance values between materials. A more precise and complete analysis which has not been implemented in this investigation, requires the use of appropriate numerical models that account for stress–strain behaviour and the accurate acoustic propagation of shock wave in the multi-layered composite. Furthermore, in order to create complete analytical or numerical models of rain droplet impact and corresponding physical rain erosion testing, it is necessary to characterise the failure resistance of the system interfaces, so that is what this work has been focussed on.

In future research, it is necessary to implement ultrasonic testing to accurately measure the impedance of materials and take into account improved models for the stress–strain behaviour in order to cover for the viscoelastic properties of the LEP systems. Nanoindentation can be a very convenient testing method to characterise such a behaviour on the interface and surface boundaries at the microscopic scale. Moreover, it can allow one to create more accurate numerical models in the future. Cohesive Zone Modelling (CZM) between layers is planned to be incorporated into the numerical modelling of the droplet impact. The input parameters for the interface CZM can be defined by means of the adhesion characterisation methodology employed in this work.

## Figures and Tables

**Table 1:** Impedance properties of selected candidate materials. Reflected and transmitted waves as a function of the incident stress at the surface (liquid–coating) and at the interface (coating–substrate).

Material Combination: Coating-Substrate	*Z_L_* (kg/m^2^s)	*Z_C_* (kg/m^2^s)	*Z_S_* (kg/m^2^s)	$\frac{\sigma_{R_{LC}}}{\sigma_{I_{LC}}}$	$\frac{\sigma_{T_{LC}}}{\sigma_{I_{LC}}}$	$\frac{\sigma_{R_{CS}}}{\sigma_{I_{CS}}}$	$\frac{\sigma_{T_{CS}}}{\sigma_{I_{CS}}}$
Gel Coat-GFRP	1.48M	3.04M	5.64M	−0.345	1.345	−0.300	1.300
LEP-GFRP	1.48M	0.09M	5.64M	0.882	0.118	−0.968	1.968

**Table 2:** Bulk properties obtained from gel coating configuration nanoindentation testing.

Material	Indentation Modulus	Hardness
Gel Coating *	6.85 ± 0.94 GPa	275.11 ± 28.08 MPa
GFRP matrix	4.64 ± 0.36 GPa	175.41 ± 9.01 MPa

* Bulk properties for Coat 1 and Coat 2 were obtained far away from the interface so as to not be influenced by the interface.

**Table 3:** Impedance properties of gel coating candidate materials. Reflected and transmitted waves as a function of the incident stress at the surface (liquid–coating) and at the interface (coating–substrate).

Material Combination: Coating–Substrate	*Z_L_* (kg/m^2^s)	*Z_C_* (kg/m^2^s)	*Z_S_* (kg/m^2^s)	$\frac{\sigma_{R_{LC}}}{\sigma_{I_{LC}}}$	$\frac{\sigma_{T_{LC}}}{\sigma_{I_{LC}}}$	$\frac{\sigma_{R_{CS}}}{\sigma_{I_{CS}}}$	$\frac{\sigma_{T_{CS}}}{\sigma_{I_{CS}}}$
Gel Coating–GFRP	1.48M	3.04M	5.65M	−0.345	1.345	−0.300	1.300
Gel Coating–GFRP Matrix *	1.48M	3.04M	2.35M	−0.345	1.345	0.128	0.872
Gel Coating *–Coat 2 interphase	1.48M	3.04M	5.22M	−0.345	1.345	−0.263	1.263
Coat 2 interphase–GFRP **		5.22M	5.65M			−0.040	1.040

* Bulk properties for Coat 1 and Coat 2 were obtained far away from the interface so as to not be influenced by the interface. Coat 2 interphase properties are only defined in the interface layer. ** The relative acoustic properties are not considered on the liquid–coating surface; the Coat 2 interface acts as a first substrate layer and the GFRP as the second substrate layer.

**Table 4:** Bulk properties obtained for LEP configuration materials with nanoindentation testing.

Material	Indentation Modulus	Hardness
LEP	21.37 ± 0.45 MPa	6.39 ± 0.8 MPa
Primer	3.66 ± 0.29 GPa	130.75 ± 47 MPa
Filler	8.76 ± 0.87 GPa	167.37 ± 14.17 MPa

**Table 5:** Impedance properties of LEP candidate materials. Reflected and transmitted waves as a function of the incident stress at the surface (liquid–coating) and at the interface (coating–substrate).

Material Combination: Coating–Substrate	*Z_L_* (kg/m^2^s)	*Z_C_* (kg/m^2^s)	*Z_S_* (kg/m^2^s)	$\frac{\sigma_{R_{LC}}}{\sigma_{I_{LC}}}$	$\frac{\sigma_{T_{LC}}}{\sigma_{I_{LC}}}$	$\frac{\sigma_{R_{CS}}}{\sigma_{I_{CS}}}$	$\frac{\sigma_{T_{CS}}}{\sigma_{I_{CS}}}$
LEP–GFRP	1.48M	0.09M	5.65M	0.882	0.118	−0.968	1.968
LEP–Filler	1.48M	0.09M	2.83M	0.882	0.118	−0.936	1.936
LEP–Primer	1.48M	0.09M	2.28M	0.882	0.118	−0.922	1.922
Primer–Filler *		2.28M	2.83M			−0.107	1.107

* The relative acoustic properties are not considered on the liquid–coating surface and the primer acts as a first substrate layer instead of the coating layer and the filler as the second substrate layer.
